# Documented rituals in pediatric intensive care: a decade of sacramental and symbolic practices in a pluralistic clinical setting

**DOI:** 10.1186/s12887-025-06430-w

**Published:** 2026-02-05

**Authors:** Steven Hébert, Heiko Reutter, Gregor Hanslik, Joachim Woelfle, Melanie L. Conrad, Fabian B. Fahlbusch

**Affiliations:** 1 Division of Neonatology and Pediatric Intensive Care Medicine, Department of Pediatrics and Adolescent Medicine, Friedrich-Alexander-Universität Erlangen-Nürnberg, Loschgestr. 15, Erlangen, 91054 Germany; 2 Department of Pediatrics and Adolescent Medicine, Friedrich-Alexander-Universität Erlangen-Nürnberg, Loschgestr. 15, Erlangen, 91054 Germany; 3 Section Neonatology and Pediatric Intensive Care, Department of Pediatrics and Adolescent Medicine Faculty of Medicine, University of Augsburg, Stenglinstr. 2, Augsburg, 86156 Germany; 4 Institute of Microbiology, Infectious Diseases and Immunology, Charité-Universitätsmedizin Berlin, Corporate Member of Freie Universität Berlin, Humboldt-Universität zu Berlin, Berlin, 12203 Germany

**Keywords:** Pediatric intensive care, Neonatal intensive care, Rituals, Sacramental practices, Symbolic practices, Staff-initiated rituals, Faith alignment

## Abstract

**Background:**

Rituals with spiritual or symbolic meaning form an integral part of pediatric intensive care, yet their timing, initiators, and contextual functions remain insufficiently described. In secularizing and religiously diverse societies, understanding how such practices are documented and enacted is essential for ethically grounded care. This study characterizes sacramental and symbolic rituals in a German tertiary neonatal and pediatric intensive care unit (NICU/PICU), examining initiation patterns, faith alignment, and survival-related timing.

**Methods:**

We conducted a retrospective descriptive analysis of 135 neonates and infants who received a documented ritual between 2013 and 2024. Rituals were categorized as sacramental or symbolic. Initiators, performers, clinical context, and survival category were recorded. Faith alignment was defined by the correspondence between family affiliation and ritual performer. Data from chaplaincy and clinical documentation were analyzed descriptively.

**Results:**

Most rituals were initiated by healthcare staff (≈ 67%) and performed by clergy (≈ 70%). Rituals occurred across the full range of survival outcomes but clustered in intermediate prognostic categories, where uncertainty was greatest. Symbolic and staff-led rituals were used predominantly in time-critical situations, particularly when clergy were unavailable or denominational alignment was unclear. Cross-faith rituals were rare and mainly observed in acute phases. Ritual–faith congruence increased with longer survival trajectories. No distinct non-sacramental religious rituals were documented, likely reflecting under-capture of informal practices.

**Conclusions:**

Ritual practice in pediatric intensive care extends well beyond last rites, encompassing symbolic, anticipatory, and adaptively tailored acts integrated into routine clinical care. These patterns reflect the influence of urgency, availability, and cultural diversity on ritual expression. Documentation gaps limit full quantification and underscore the need for prospective, mixed-methods studies and inclusive institutional frameworks for culturally and spiritually responsive care.

## Background

In neonatal and pediatric intensive care, the end of life often unfolds under conditions of emotional and cultural complexity. Families frequently turn to spiritual or symbolic rituals - such as baptism, blessings, or personalized symbolic acts - to create meaning, support decision-making, and maintain a sense of connection. These practices mediate the space between medical intervention and family identity, yet their implementation, timing, and documentation remain poorly characterized, particularly in pluralistic and secularizing societies.

Within ritual studies, rituals are understood as structured, embodied actions that convey meaning through symbolic performance rather than verbal expression alone [1–3]. In clinical settings, such acts often emerge at moments of uncertainty, transition, or loss. Importantly, rituals in this conceptual sense are not limited to end-of-life contexts; they may occur at any point along the illness trajectory when families and clinicians face ambiguity, vulnerability, or the need for symbolic action. Drawing on this framework, spiritual and symbolic practices can be analyzed not only by their theological content but also by their function, degree of formalization, and relational context.

For conceptual orientation, ritual practices in intensive care can be understood as spanning a spectrum from formal, theologically grounded rites to more flexible symbolic acts created by families or staff. Sacramental rituals, non-sacramental religious practices, and individualized symbolic acts each serve distinct functions in expressing identity, continuity, or transition. In pluralistic clinical settings, these forms frequently coexist and may be adapted pragmatically to clinical circumstances. Because many symbolic or informal practices occur spontaneously at the bedside, retrospective documentation may capture only part of this spectrum.

### Psychological meaning and legacy

Rituals and symbolic acts support families’ efforts to construct meaning and preserve connection during and after a child’s critical illness or death. They enable parents to enact their caregiving role within highly medicalized environments and create tangible memories that support psychological integration of loss [4–6]. Simple acts - such as bathing, wrapping, lighting candles, or creating keepsakes - often carry deep emotional significance. Many such practices occur informally at the bedside and may not be systematically recorded, contributing to underdocumentation in retrospective analyses.

### Cultural and communal dimensions

Rituals also affirm cultural identity and communal belonging. Diverse traditions view specific rites as essential to a “good death” or meaningful transition [7]. When families are unable to access culturally congruent practices due to institutional or logistical constraints, they may experience the death as incomplete or misaligned with their values [8–10]. In multicultural clinical settings, such variability underscores the need for flexible, inclusive approaches that accommodate heterogeneous beliefs. Many culturally embedded or family-led symbolic acts fall outside formal documentation systems, further complicating quantitative assessment.

### Ethical and relational aspects

The ethical imperative to honor a family’s cultural and spiritual values is grounded in respect for autonomy, beneficence, and relational integrity. Spiritual or symbolic acts require consent, cultural sensitivity, and attentiveness to denominational alignment. When clergy access is limited or when families’ affiliations are unclear, clinicians may support families in creating staff-led or interfaith symbolic acts, which can alleviate distress and strengthen relational continuity [4, 5]. Such practices carry emotional and ethical significance for both families and clinicians and may influence how they navigate decision-making, dying, and memory-making.

### Rituals in homogeneous vs. pluralistic clinical contexts

The study by Caulfield et al. [11] provides insight into ritual practice within a mono-denominational Catholic NICU, where emergency baptism served as the dominant sacramental response to clinical deterioration. In that cohort, early baptisms closely tracked neonatal mortality patterns and reflected a theologically cohesive ritual environment. While this model illustrates the ritual logic of a culturally uniform setting, it does not address how rituals function in pluralistic, secular, or multifaith contexts - nor how rituals are adapted when denominational alignment cannot be guaranteed.

### Gaps and study rationale

Despite their importance for families and clinical teams, ritual practices in contemporary intensive care have not been systematically examined in pluralistic environments. Existing studies have focused primarily on Christian sacramental rites, with limited attention to symbolic or cross-faith practices, staff-initiated rituals, or timing relative to survival trajectories. Furthermore, the degree to which these practices are documented - particularly informal or post-mortem rituals - remains unclear. The present study addresses these gaps by characterizing sacramental and symbolic rituals documented over a ten-year period in a culturally diverse NICU/PICU. We analyze (1) ritual typology, (2) initiation and performance patterns, (3) faith alignment, and (4) timing relative to survival categories. By situating ritual practice within a pluralistic clinical environment, this study seeks to clarify how families and clinicians use symbolic and sacramental acts across the illness trajectory and to inform more inclusive institutional frameworks for spiritual and symbolic care.

## Methods

### Study design and setting

We conducted a retrospective, single-center descriptive study at the neonatal and pediatric intensive care units (NICU/PICU) of the University Children’s Hospital Erlangen, Germany. The observation period covered all admissions between October 2013 and December 2024. The institution includes an integrated pediatric cardiac center, providing comprehensive in-house management for neonates and infants with congenital heart disease (CHD), including stabilization, cardiac surgery, and postoperative intensive care. This configuration results in a clinically heterogeneous patient population with a substantial proportion of complex CHD and explains the presence of early deaths in several cases. Both inborn patients and those transferred from regional and supraregional hospitals were included. The study aimed to describe the implementation, timing, and contextual characteristics of ritual practices performed for critically ill neonates and infants within this clinical environment. No inferential comparisons or outcome analyses were planned.

### Identification of ritual events and data sources

Ritual events were identified through a joint chaplaincy–clinical register maintained by hospital chaplains and attending physicians. Entries included the date and type of ritual, initiator, performer, location, and brief contextual notes. Additional clinical information (diagnosis, demographic data, survival) was retrieved from electronic medical records (Soarian by Cerner; ICM by Dräger) and cross-checked with discharge or death summaries. Because the chaplaincy register was the primary source of ritual documentation, informal or spontaneous symbolic acts may not have been recorded, representing a structural limitation acknowledged in this study.

### Definitions and categorization of rituals

Rituals were defined as structured, embodied acts with spiritual or symbolic significance enacted by families, clergy, or healthcare professionals. Based on description, performer identity, and contextual notes, each documented act was assigned to one of three mutually exclusive categories:

- Sacramental rituals: formal, theologically codified rites performed by ordained clergy (e.g., baptism, anointing).
- Non-sacramental religious rituals: explicitly religious practices without sacramental status *(e.g.*,* blessings*,* prayers).* No such practices were documented in this cohort.
- Symbolic acts: non-doctrinal gestures conveying emotional or symbolic meaning (e.g., bathing rituals, candle lighting, hand molds).

Two investigators (FBF and SH) independently classified each act; discrepancies were resolved by consensus. Faith alignment was defined as correspondence between the family’s stated religious affiliation and the ritual performer (aligned / non-aligned / unspecified).

### Study population and inclusion criteria

All neonates and infants admitted to the NICU or PICU during the study period who received at least one documented ritual act were included. Each patient was counted once; when multiple rituals were present, only the first was analyzed to standardize timing metrics.

### Variables collected

Demographic and clinical variables: date of birth, sex, gestational age; primary diagnosis group; care setting (NICU or PICU); survival status and age at death or last follow-up. Ritual characteristics: ritual category (sacramental / symbolic / non-sacramental religious); initiator (e.g., physician, parent, nurse); performer (clergy, staff, family); location; faith alignment.

### Timing measures

For descriptive temporal analysis, we extracted: days from birth to first documented family counseling regarding end-of-life or critical decisions; days from birth to ritual; days between counseling and ritual; days from ritual to death (if applicable); age at death. Large outliers (e.g., prolonged hospitalizations or later follow-up encounters) were retained to reflect the underlying clinical heterogeneity.

### Survival categories

To describe the temporal relationship between ritual timing and clinical course, each patient was assigned to one of seven mutually exclusive survival categories based on postnatal age at death or survival beyond 6 months: Category 0: day 0; Category 1: days 1–7; Category 2: days 8–30; Category 3: days 31–180; Category 4: days 181–365; Category 5: >365 days; Category 6: survivors (alive at ≥ 6 months of follow-up). The categories reflect clinically recognized developmental epochs and ensured sufficient distribution across categories. These groupings were used for descriptive purposes only; no inferential analyses were performed.

### Diagnostic groupings

Primary diagnoses were categorized into: congenital heart disease (CHD); prematurity, including extreme prematurity; perinatal asphyxia; genetic or syndromic disorders; metabolic diseases; oncologic diseases; other diagnoses (infectious, neurologic, or surgical conditions).

### Statistical analysis

Data were organized using MS Office and analyzed descriptively in GraphPad Prism (v10.4.1, GraphPad Software, Boston, MA, USA). Continuous variables are reported as medians with interquartile ranges (IQR), reflecting the skewed distribution of timing measures in this cohort. Categorical variables are presented as counts and percentages. No hypothesis testing or inferential comparisons were conducted due to cohort heterogeneity and the contextual nature of ritual practices.

### Demographic context

To contextualize religious and cultural diversity, municipal and regional demographic data were retrieved from official statistical sources for the Administrative Region of Central Franconia [12, 13] and the city of Erlangen [14], Germany. The University Children’s Hospital Erlangen serves a culturally and religiously diverse urban population. As of 2025, 25% of Erlangen’s 120,028 residents hold foreign citizenship, and approximately 40% identify as first-, second-, or third-generation migrants [14]. Religious affiliation is heterogeneous: 21.7% of residents identify as Protestant and 21.3% as Roman Catholic, while the remaining ~ 57% belong to minority religious communities or report no religious affiliation. The Administrative Region of Central Franconia, from which Erlangen draws a substantial proportion of referrals, reflects a similar pattern, with more than 40% of residents unaffiliated or belonging to non-majority traditions [12, 13]. This pluralistic population structure directly informs the diversity of spiritual backgrounds encountered in the NICU/PICU and provides the contextual foundation for the range of sacramental, symbolic, and cross-faith ritual practices observed in this study. It also highlights the likelihood of informal, family-specific practices that may not be fully captured in retrospective documentation systems.

## Results

### Study population

During the ten-year observation period, 508 neonates and infants died in the NICU or PICU. Among these, 135 patients (26.6%) had at least one documented ritual act and therefore formed the study cohort. The remaining 402 patients had no documented ritual in the available chaplaincy or clinical records. Absence of documentation should not be interpreted as absence of spiritual or symbolic care, given the likelihood of undocumented informal practices. The cohort reflects the religious and cultural diversity of the institution’s catchment area (see Methods).

Rituals included sacramental-canonical rites (e.g., baptisms), symbolic acts without formal theological reference, and no documented non-sacramental religious rituals. Of the 135 patients receiving a ritual, 29 (21.5%) survived to discharge or later follow-up. All deaths in the ritual cohort occurred during the index hospitalization.

### Diagnostic groups

**Fig. 1:**
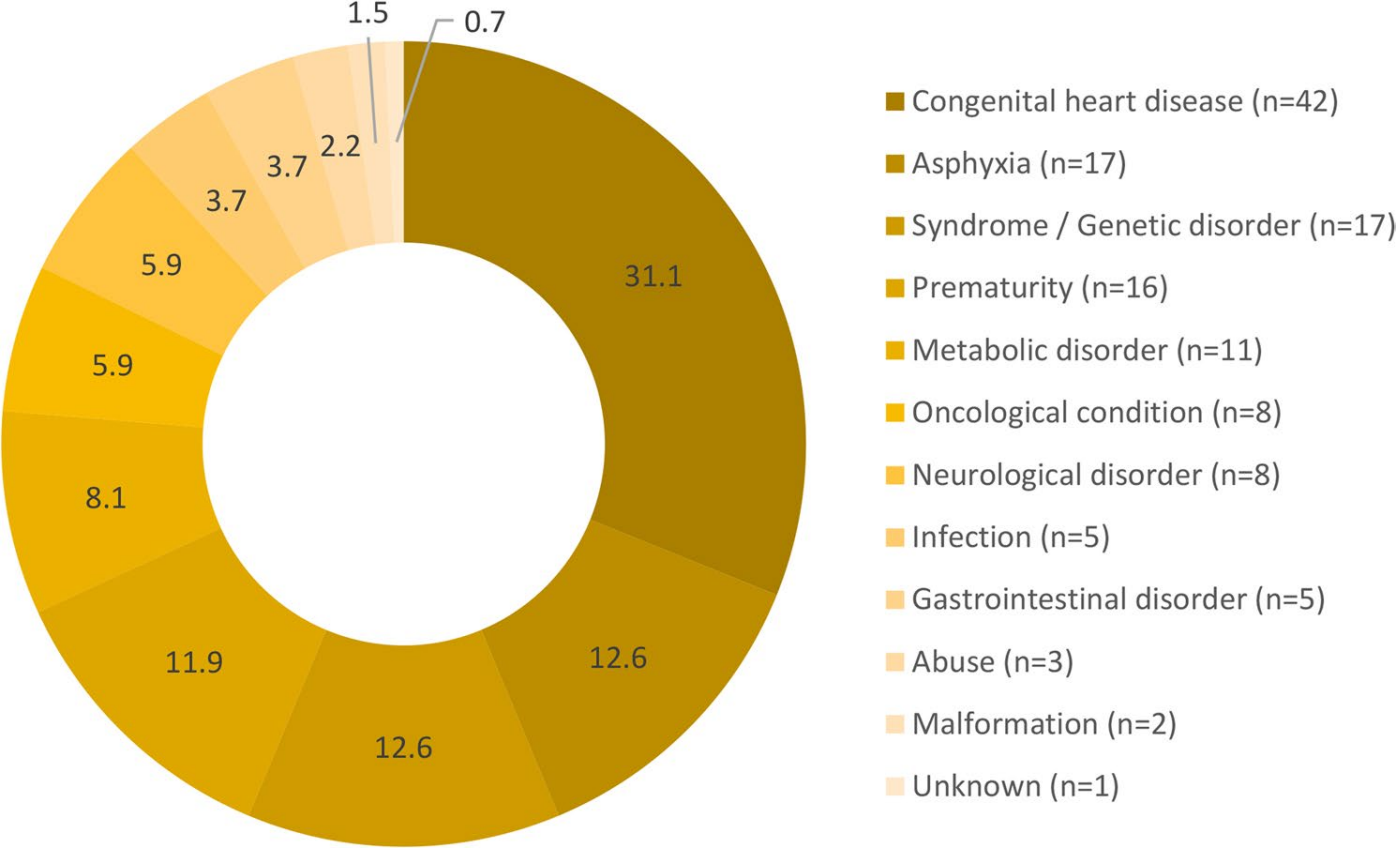
Primary diagnoses among patients receiving ritual acts (*n* = 135). Proportional distribution (%) and absolute numbers are shown. CHD was the largest subgroup, followed by perinatal asphyxia, prematurity, genetic/syndromic disorders, metabolic and oncologic conditions, and other diagnoses

The distribution of primary diagnoses among patients receiving ritual acts is shown in Fig. 1. Congenital heart disease (CHD) was the largest subgroup (31.1%), followed by perinatal asphyxia and genetic or syndromic disorders (12.6%), prematurity including extreme prematurity (11.9%), metabolic disorders (7.4%), and oncologic conditions (4.4%). The remaining 17.8% comprised a heterogeneous group of infectious, neurologic, or surgical conditions. The diagnostic spectrum reflects the heterogeneous case mix typical of a tertiary NICU/PICU with a pediatric cardiac center. Figure 1 displays the proportional distribution of diagnostic groups within survival categories and is not intended to represent disease-specific mortality.

### Ritual distribution across care settings

**Fig. 2:**
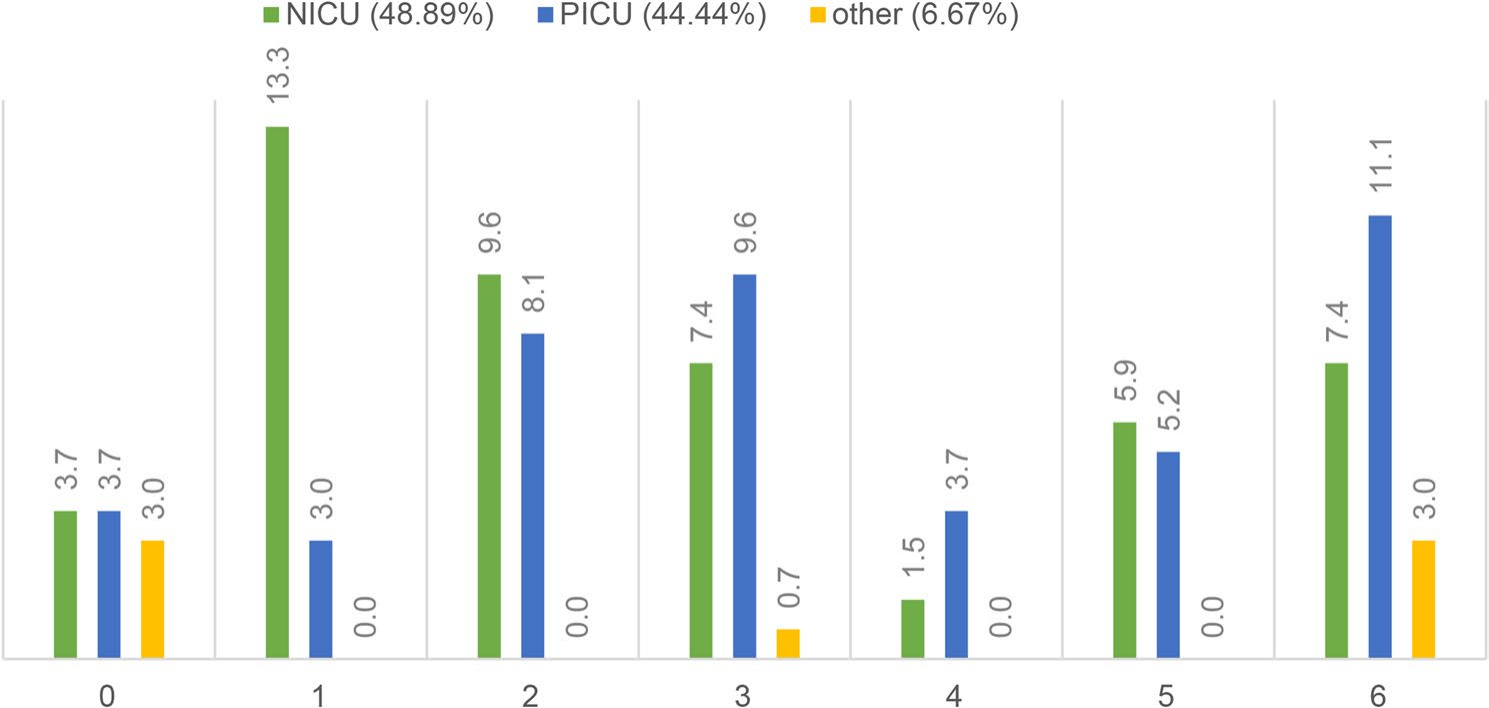
Distribution of rituals across survival categories (0–6) stratified by care setting. Relative frequencies (%) are shown for the NICU and PICU. “Other” includes delivery room, obstetrics, and peripheral ward locations

Approximately half of all rituals were performed in the NICU (48.9%) and half in the PICU (44.4%), with 6.7% performed in adjacent units (delivery room, obstetric ward). Rituals occurred across all survival categories in both settings (Fig. 2). In the NICU, rituals were more frequent in early survival categories (0–1), while the PICU showed a higher proportion in long-term survivors (category 6), consistent with differences in case mix and age distribution.

### Timing of counseling, rituals, and death

Timing metrics are summarized in Table 1. In several cases, the ritual occurred before the first documented counseling entry, which explains why the median age at ritual was lower than the median age at counseling. The ritual was typically performed shortly after the documented counseling conversation (median delay 0 days, IQR 0–1). The age at death showed substantial variability (median 20 days, IQR 4-121), reflecting heterogeneous clinical trajectories. Individual outliers, including long-term survivors who later died during prolonged admission or follow-up encounters, account for extended ranges (e.g., > 1000 days). These values were retained to reflect the underlying clinical spectrum.

**Table 1:** Timing of family counseling, ritual performance, and death

Metric	Median (Range)	IQR (25–75%)
Days to counseling	25 (0-4175)	3-115
Days to ritual	17 (0-4748)	3-116.5
Days between counseling and ritual	0 (0–17)	0–1
Days from ritual to death	1 (0-636)	0–8
Age at death (days)	20 (0-4749)	4-121

Medians with interquartile ranges (IQR) are shown. Wide ranges reflect heterogeneous clinical trajectories within the cohort

To describe temporal patterns relative to clinical course, patients were grouped into the seven survival categories defined in the Methods (Categories 0–6: day 0; days 1–7; 8–30; 31–180; 181–365; >365 days; survivors at ≥ 6 months). Category-specific timing metrics are shown in Table 2. Earlier categories (0–1) showed short intervals from birth to ritual and from ritual to death, reflecting acute neonatal conditions. In categories 3–5, longer intervals corresponded to more prolonged illness courses. Category 6 comprised survivors at ≥ 6 months of follow-up. Across categories, ritual timing did not exhibit a monotonic relationship with survival; instead, rituals clustered in intermediate categories (2–3), representing periods of heightened prognostic uncertainty.

**Table 2:** Timing metrics by survival category

Survival Category	Days to counseling (Median, IQR)	Days to ritual (Median, IQR)	Days between counseling and ritual (Median, IQR)	Days from ritual to death (Median, IQR)	Age at death (days) (Median, IQR)
0	0 (0–0)	0 (0–0)	0 (0–0)	0 (0–0)	0 (0–0)
1	1 (1–2.5)	2 (1.5–3)	0 (0–1)	0 (0–1)	4 (2–4.75)
2	8 (4.25–15)	9.5 (3.75–17)	0 (0–1)	3 (–1–5.25)*	16 (12–20.5)
3	54.5 (50–94.75)	54.5 (25.5–82.5)	0 (0–1)	6 (0–12.75)	66 (54–105.25)
4	191 (182.5–203)	191 (183–205.5)	0 (0–1)	1 (0–3.5)	222 (193–228)
5	1461 (406–2295)	1461 (390–2433.5)	0 (0–1)	5 (0–22)	1463 (631–2568)
6 (survivors)	32 (7.5–210.5)	47 (9–682.5)	1 (0–2)	n/a	n/a

Survival categories (0–6) were defined according to postnatal age at death or survival at ≥ 6 months. Values represent medians with interquartile ranges (IQR)

Legend: *Lower bounds below zero indicate highly skewed distributions in early categories with very small median values. *n/a* not applicable

### Distribution of ritual categories and faith alignment

**Fig. 3:**
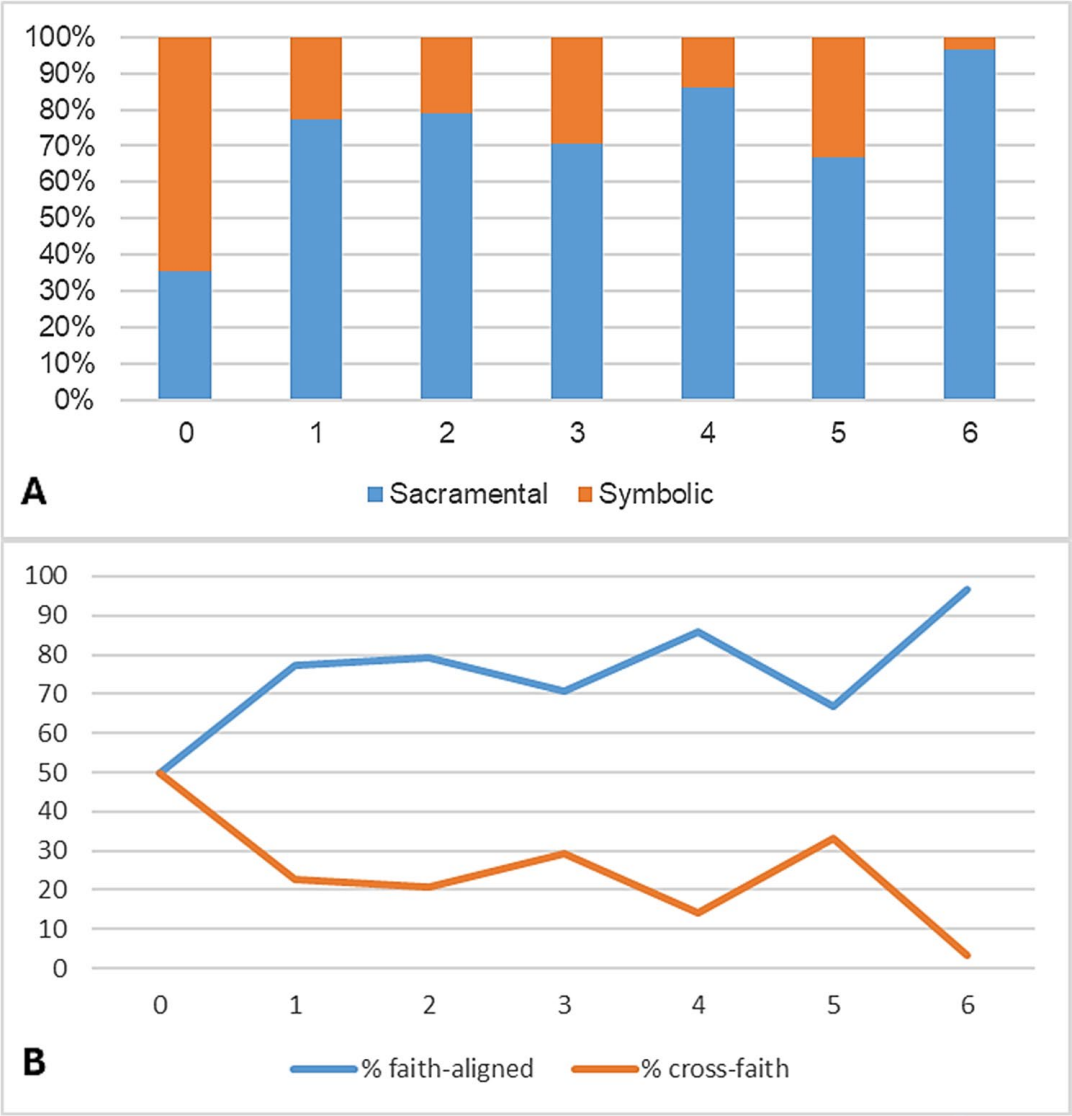
**A** Distribution of ritual types across survival categories. Rituals were categorized as sacramental or symbolic. No non-sacramental religious rituals were documented. **B** Faith alignment across survival categories. Rituals were classified as faith-aligned, cross-faith, or unspecified based on correspondence between the family’s stated affiliation and the ritual performer

Across the cohort, sacramental-canonical rituals accounted for 75.5% and symbolic acts for 24.4% of documented rituals (Fig. 3A). No non-sacramental religious rituals were recorded, likely reflecting documentation practices rather than true absence.

Rituals were documented in all survival categories. Sacramental rituals were most frequent in categories 1–3 and increased again among survivors (category 6). Symbolic acts were more common in categories 0–2, reflecting their flexible use in acute or time-critical settings.

Faith alignment is shown in Fig. 3B. Most rituals (≈ 82%) were performed within the family’s stated religious tradition. Cross-faith rituals accounted for ≈ 14% and occurred predominantly in early, acute categories (0–1). Ritual-faith alignment increased with longer survival: in categories 5–6, nearly all rituals were aligned.

### Initiators and performers of rituals

Initiator and performer distributions are presented in Table 3. Most rituals were initiated by healthcare professionals (≈ 67%), followed by parents/family (≈ 29%), with few initiated by others (≈ 3%). Staff initiation predominated across all survival categories, whereas parent initiation increased with longer survival.

Regarding performers, ordained clergy conducted most rituals. Staff-performed rituals - by physicians, nurses, or midwives - were uncommon overall but present in early categories, particularly category 0, reflecting their involvement in time-sensitive or birth-related contexts. Family clergy appeared infrequently.

**Table 3:**
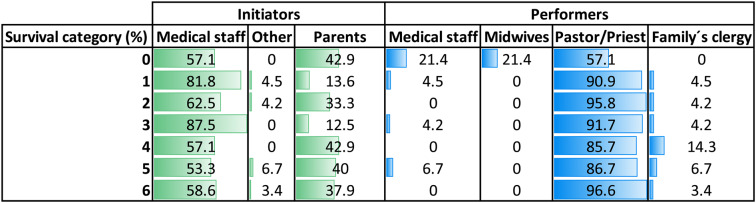
Initiators and performers of rituals across survival categories

Distribution of initiators (green) and performers (blue) among all documented ritual acts (*n* = 135). Percentages of documented ritual initiators and performers are shown for each survival category (0–6). Initiator categories include *medical staff* (physicians or senior residents), *parents*, and other (grandparents, legal guardians, or unspecified). Performer categories include *medical staff* (physicians or senior residents), *midwives, parents, pastor/priest* (institutional clergy), and *family’s clergy* (external or home priest)

## Discussion

### Summary of main findings

In this ten-year descriptive analysis of 135 documented rituals in a tertiary NICU/PICU, ritual practice was observed across the full continuum of clinical trajectories. Three consistent patterns emerged. First, rituals clustered in intermediate prognostic categories, indicating that ritual practice was not restricted to the terminal phase but also occurred during periods of prognostic uncertainty. Second, most rituals were initiated by healthcare staff, underscoring that ritual practice is structurally embedded in intensive care and not exclusively family- or clergy-driven. Third, symbolic acts predominated in acute or time-critical contexts, whereas sacramental rituals became more common with longer survival. Faith alignment increased with survival time, and cross-faith rituals occurred primarily in early, acute categories. No non-sacramental religious rituals were documented, likely reflecting limitations in retrospective recording rather than true absence. Together, these findings depict ritual practice as a flexible component of intensive care that adapts to urgency, availability, and cultural context.

### Ritual timing and trajectories in context

Prior studies, such as the mono-denominational Catholic cohort described by Caulfield et al. [11], have associated neonatal baptisms predominantly with imminent or early death. In contrast, the present cohort demonstrates that rituals in contemporary, pluralistic intensive care settings occur throughout the illness trajectory, with a notable concentration in mid-course clinical phases. These phases often involve evolving prognostic information, uncertain decision points, and complex family communication, which may prompt ritual engagement independently of the proximity to death.

The median timing and broad ranges observed across survival categories reflect heterogeneous illness courses typical of tertiary intensive care. Early rituals were common in conditions characterized by rapid deterioration, whereas rituals in longer survival categories occurred after extended stabilization or recovery. This distribution aligns with literature suggesting that ritual practice supports families not only at the end of life but also during moments of transition, decision-making, and emotional disequilibrium [4–6].

### Initiators, performers, and the role of clinical teams

Across all survival categories, healthcare professionals were the most frequent initiators of ritual acts. This finding is consistent with reports that clinicians often identify moments of heightened emotional or ethical importance and support families in accessing or creating ritual practices [4, 5]. In the NICU, staff-initiated and staff-performed rituals were particularly prominent in early categories, where rapid clinical deterioration and limited clergy availability require time-sensitive responses. Notably, midwives appeared as performers alongside physicians and nurses, especially during birth-related or immediate postnatal contexts. Their involvement reflects the relational continuity they provide at the intersection of birth, acute stabilization, and end-of-life care, a contribution emphasized in recent literature on perinatal bereavement support and psychosocial care [15, 16]. The predominance of clergy as ritual performers underscores the continued relevance of formal spiritual roles within pediatric intensive care. However, the presence of staff-performed and family-performed symbolic acts highlights that ritual practice extends beyond sacramental frameworks and incorporates flexible, relational gestures that respond to urgency, cultural diversity, and family preference.

### Symbolic, sacramental, and cross-faith practices

Symbolic rituals were more common in early and acute categories, consistent with their adaptability when denominational alignment is uncertain or clergy availability is limited. Sacramental rituals predominated in categories 1–3 and increased again among survivors, suggesting both acute and planned uses. The near absence of documented non-sacramental religious rituals likely reflects documentation practices, as informal blessings, prayers, or symbolic actions may not be consistently recorded in chaplaincy registers. Faith alignment increased with longer survival, indicating that families with more extended clinical trajectories were more often able to access clergy from their own tradition. Cross-faith rituals, though infrequent, occurred mainly in categories 0–1 and appear to represent pragmatic responses to time pressure and limited denominational matching during acute care, rather than deliberate exclusion or neglect. As described by Kobler et al. [4] and Thorvilson et al. [7], mismatches between a family’s spiritual identity and the ritual provided may nonetheless contribute to disenfranchised grief or a sense of spiritual incompleteness. Ensuring culturally sensitive pathways - including timely access to clergy from diverse traditions and staff trained in interfaith or non-denominational symbolic support - may help mitigate these risks. Such frameworks allow institutions to respond to spiritual needs without defaulting to a single denominational model, particularly in rapidly evolving or time-limited clinical situations.

### Documentation and structural limitations

This analysis relies on retrospective documentation, which introduces several inherent constraints. Chaplaincy registers are not designed to capture informal, spontaneous, or family-led actions, nor do they consistently record post-mortem practices such as ritual washing, bathing, dressing, or other culturally meaningful preparations of the body. As a result, the dataset likely underrepresents symbolic practices, particularly those performed at the bedside without clergy involvement, during acute deterioration, or after death. In addition, broader demographic trends - such as the well-documented decline in traditional religious affiliation in Germany and increasing cultural diversity in the region—may further contribute to a higher prevalence of individualized or symbolic practices that are less consistently recorded in chaplaincy systems. The absence of documented non-sacramental religious rituals in this cohort is best interpreted as a consequence of documentation patterns rather than a true absence of such practices in clinical care. Documentation rarely included details about families’ motivations, emotional context, or cultural framing of rituals, and parental perspectives were not available. Transfers within the hospital or to external institutions may also have contributed to under-capture. Moreover, the descriptive study design precludes causal inference, and findings reflect practices within a single tertiary center with a specific demographic profile. Prospective mixed-methods designs, including structured observation and family or staff interviews, are needed to capture the full scale of ritual practice and its ethical and emotional dimensions.

### Implications for practice

These findings have several implications for clinical practice. Pluralistic, diverse care settings require flexible institutional pathways that allow families to access clergy or spiritual representatives from their own traditions when feasible. Structured support for symbolic and staff-facilitated rituals, including in non-terminal phases, may benefit families navigating prognostic uncertainty. Staff training that includes awareness of ritual diversity, communication strategies, and consent considerations may facilitate culturally responsive care. Systematic documentation, whether in PDMS systems or standardized registries, could enhance continuity, transparency, and quality improvement efforts. Contemporary models of pediatric palliative and anticipatory care increasingly emphasize relational presence, emotional continuity, and attention to family values [17]. Integrating ritual practice into interdisciplinary workflows can help align care with family needs across the illness trajectory and may support both families and staff as they navigate ethically and emotionally complex situations.

### Strengths and limitations

Strengths of this study include its decade-long observation period, inclusion of both NICU and PICU settings, and systematic categorization of ritual acts across survival categories. The pluralistic clinical context provides insight into ritual practice outside mono-denominational environments and contributes evidence from a setting where the cultural and religious landscape is shifting.

Several limitations must be considered. This study relies on retrospective documentation, primarily from a chaplaincy-based register that is not designed to capture informal, spontaneous, or family-led actions. As a result, the dataset likely underrepresents symbolic practices, particularly those performed at the bedside without clergy involvement, during acute deterioration, or as post-mortem acts (e.g., bathing, dressing, washing, or other culturally meaningful preparations of the body). The absence of documented non-sacramental religious rituals in this cohort is therefore best interpreted as a consequence of documentation patterns rather than a true absence of such practices in clinical care.

Documentation rarely included details about families’ motivations, emotional context, or cultural framing of rituals, and parental perspectives were not available. Transfers within the hospital or to external institutions may have contributed to additional under-capture. Moreover, the descriptive study design precludes causal inference, and findings reflect practices within a single tertiary center with a specific demographic profile. Prospective, mixed-methods designs incorporating direct observation and family and staff interviews are needed to capture the full breadth of ritual practice and its ethical and emotional dimensions.

## Conclusion

Ritual practice in neonatal and pediatric intensive care is diverse, adaptive, and integrated into clinical care across the illness trajectory. Rituals occur not only at the end of life but also during periods of prognostic uncertainty, often initiated by clinical teams and shaped by the pluralistic context of contemporary intensive care. Improving documentation and supporting flexible, culturally responsive ritual practices may enhance family experience and ethical care in the NICU and PICU.

## Data Availability

All necessary data are included within the manuscript. Additional data supporting the findings of this study are available from the corresponding author upon reasonable request. Ritual documentation cannot be publicly shared due to the sensitive nature of clinical and pastoral records.
